# Dendritic Architecture Predicts *in vivo* Firing Pattern in Mouse Ventral Tegmental Area and Substantia Nigra Dopaminergic Neurons

**DOI:** 10.3389/fncir.2021.769342

**Published:** 2021-11-19

**Authors:** Trinidad Montero, Rafael Ignacio Gatica, Navid Farassat, Rodrigo Meza, Cristian González-Cabrera, Jochen Roeper, Pablo Henny

**Affiliations:** ^1^Laboratorio de Neuroanatomía, Departamento de Anatomía, and Centro Interdisciplinario de Neurociencia, NeuroUC, Escuela de Medicina, Pontificia Universidad Católica de Chile, Santiago, Chile; ^2^Institute of Neurophysiology, Goethe University, Frankfurt, Germany

**Keywords:** dopamine, substantia nigra, ventral tegmental area, dendritic morphology, firing properties, neuronal tracing

## Abstract

The firing activity of ventral tegmental area (VTA) and substantia nigra pars compacta (SNc) dopaminergic (DA) neurons is an important factor in shaping DA release and its role in motivated behavior. Dendrites in DA neurons are the main postsynaptic compartment and, along with cell body and axon initial segment, contribute to action potential generation and firing pattern. In this study, the organization of the dendritic domain in individual VTA and SNc DA neurons of adult male mice, and their relationship to *in vivo* spontaneous firing, are described. In comparison with dorsal VTA DA neurons, ventrally located VTA neurons (as measured by cell body location) possess a shorter total dendritic length and simpler dendritic architecture, and exhibit the most irregular *in vivo* firing patterns among DA neurons. In contrast, for DA neurons in the SNc, the higher irregularity of firing was related to a smaller dendritic domain, as measured by convex hull volumes. However, firing properties were also related to the specific regional distribution of the dendritic tree. Thus, VTA DA neurons with a larger extension of their dendritic tree within the parabrachial pigmented (PBP) nucleus fired more regularly compared with those with relatively more dendrites extending outside the PBP. For DA neurons in the SNc, enhanced firing irregularity was associated with a smaller proportion of dendrites penetrating the substantia nigra pars reticulata. These results suggest that differences in dendritic morphology contribute to the *in vivo* firing properties of individual DA neurons, and that the existence of region-specific synaptic connectivity rules that shape firing diversity.

## Introduction

Dopaminergic neurons of the substantia nigra pars compacta (SNc) and ventral tegmental area (VTA) are involved in important brain functions, such as movement, behavioral reinforcement, and learning (Wise, 2004; Schultz, 2007; Fahn, 2008; Redgrave et al., 2008; Berke, 2018). Dopaminergic (DA) neurons exhibit an autonomous, pacemaker-type of firing that depends on intrinsic membrane properties and is typically responsible for the observed regular or tonic firing of DA neurons in the absence of synaptic input (Gantz et al., 2018). On the other hand, excitatory synaptic activity can lead to phasic and/or irregular firing, as typically observed *in vivo*, and, conversely, afferent inhibitory activity produces a decrease or halt in firing (Paladini and Roeper, 2014; Gantz et al., 2018). While tonic, regular activity contributes to the maintenance of a basal level of dopamine release in targeted regions such as the striatum (Schultz, 2007; Sulzer et al., 2016), phasic increase or decrease in DA release, on the other hand, may allow for plastic changes in postsynaptic structures in association to preference or avoidance learning (Schultz, 2007; Chang et al., 2015, 2018).

Anatomical studies demonstrated that in SNc and VTA DA neurons the vast majority of synaptic inputs occurs in the dendritic field (Rinvik and Grofová, 1970; Bolam and Smith, 1990; Bayer and Pickel, 1991; Henny et al., 2012) as compared with less frequent innervation of somatic (Rinvik and Grofová, 1970) or axon initial segment (González-Cabrera et al., 2017) compartments. This is also in line with evidence from single cell reconstructions that show dendrites account for up to 90% of the somatodendritic surface in nigral neurons (Meza et al., 2018). Considering the role that synaptic inputs play in the firing behavior of DA neurons, understanding how the dendritic domain is organized in this population and how it relates to single cell behavior *in vivo* is a critical issue to examine.

The role that dendrites, more specifically dendritic spatial organization, plays in the *in vivo* firing behavior of DA neurons has only been partially examined. For instance, we have previously shown that in rat SNc DA neurons the likelihood of firing to decrease or pause after nociceptive stimulation is associated with the extension of dendrites that penetrate the underlying substantia nigra pars reticulata (SNr) (Henny et al., 2012). Because SNr penetrating dendrites receive a much denser afferent inhibitory innervation than those located in the SNc, they may be thought as a distinctive compartment involved in mediating inhibition (Hajós and Greenfield, 1994; Henny et al., 2012). We have also examined recently the role that the somatic and dendritic domains play in *in vivo* spontaneous firing frequency in mouse nigral DA neurons and found that, in this case, spontaneous firing frequency did not seem to depend strongly on somatodendritic surface (the vast majority of which corresponds to dendrites, as indicated above). In contrast, the firing rate is related much more strongly with axon initial segment size or position (Meza et al., 2018).

With these antecedents in mind, we set out a study to (1) quantitatively describe the dendritic organization of mouse SNc and VTA DA neurons at the individual cell level, with special attention to the course of dendrites across substantia nigra and VTA regional subdivisions (Fu et al., 2012) and to (2) assess the relationship between the dendritic organization and firing pattern. For that, we performed *in vivo* juxtacellular labeling and vector-based 3D reconstruction (Henny et al., 2014; Meza et al., 2018; Farassat et al., 2019) of the complete somatodendritic domain of 15 SNc and 15 VTA DA neurons of adult mice, and analyzed in 25 of these neurons spontaneous firing rate and firing pattern.

Our results suggest that individual neuron morphology and heterotopic extension of dendrites across adjacent anatomical subdivisions within substantia nigra and VTA play an important role in shaping *in vivo* firing pattern, and suggest region-specific anatomical rules underlying firing diversity.

## Materials and Methods

### Animals

The reported experimental procedures and results were obtained from 25 adult male C57BL/6J mice. The animals were obtained from the animal house at the Faculty of Biological Sciences, Pontificia Universidad Católica de Chile, and were approved by the Ethics Committees of the School of Medicine of the Pontificia Universidad Católica de Chile which conform to the guidelines of the Comisión Nacional de Investigación Científica y Tecnológica (CONICYT) and the United States National Institutes of Health (NIH). Experimental procedures for a further five adult male C57BL/6N mice were performed at Goethe University, Germany and approved by German Regierungspraesidium Darmstadt (V54-19c20/15-F40/28), as also reported in Farassat et al. (2019).

### Recording and Labeling of Single Neurons

Ten SNc neuronal reconstructions came from a pool of neurons that had their electrophysiology, general dendritic arrangement, and axon initial segment described (Meza et al., 2018), and were selected for this study because their mediolateral (ML), dorsoventral (DV), and anteroposterior (AP) localization within the SNc could be unambiguously determined, thus making them suitable for the localization analysis reported here. Additionally, using the same methodological approach, two recently labeled and reconstructed SNc DA neurons from two adult mice (23–30 g) were added. These 12 SNc neurons were used in this study for anatomical and physiological analysis. Three further SNc DA neurons came from a pool of neurons recently characterized (Farassat et al., 2019) and were selected only for the anatomical analysis included in this study based on the quality and completeness of their dendritic labeling, which allowed for a complete reconstruction of their somatodendritic domain. As a result, 12 SNc neurons were used for anatomical and physiological analysis (see below), and three others for purely anatomical analysis. Regarding the VTA, 13 neurons were recorded, labeled, and completely filled with tracer, and their somatodendritic domain was 3D reconstructed. These 13 neurons were used in this study for anatomical and physiological analysis. Two further VTA DA neurons came from a pool of neurons recently characterized (Farassat et al., 2019) and were deemed suitable for a complete reconstruction of their dendritic tree by the quality and completeness of their dendritic labeling.

Anesthesia was initially induced with isoflurane (Isoflurano USP; Baxter Healthcare, USA) and maintained with urethane (1.5 g per kg, i.p., ethyl carbamate; Sigma, Germany). The animals were placed in a rat stereotaxic frame adapted to mice using a MA-6N head-holding adaptor (Narishige, Japan). Body temperature was maintained at 37°C using a homeothermic heating device (ATC 1000; World Precision Instruments, USA). Anesthesia levels were assessed by examination of the electrocorticogram (ECoG) and by testing reflexes to cutaneous pinch or gentle corneal stimulation. Topical benzocaine (20%, Mayon) and a PBS solution with pH 7.4 were applied to all surgical incisions to prevent pain and dehydration, respectively. Extracellular recordings of single-unit activity were made using borosilicate glass electrodes (1–1.5 μm diameter, tip resistance 10–15 MΩ or < 1 μm diameter and tip resistance 35–45 MΩ depending on the labeling method used (see below); World Precision Instruments), and obtained using a vertical puller (PC-10 model; Narishige Scientific Instrument Laboratory, Japan). Pipettes were filled with a solution consisting of 0.5 M NaCl or 0.25 M K + -gluconate and 1.7% neurobiotin (w/v; Vector Laboratories, USA). A single-axis *in vivo* micromanipulator (IVM-1000; Scientifica, United Kingdom) connected to an ultralow noise IU controller rack was used to descend electrodes in the z-axis into the brain. Stereotaxic coordinates for VTA single-unit recording were derived from Franklin and Paxinos (2007) (AP: −3.1 mm, ML: 0.4 mm, see Meza et al. (2018) for the description of experiments for SNc DA neurons labeling). Following single cell recordings, the neurons were labeled with the juxtacellular method (Pinault, 1996) or through intracellular access. Briefly, in the first method, the electrode was advanced slowly toward the neurons while a microiontophoretic square current was applied (2–10 nA positive current, 200 ms duration, 50% duty cycle). The optimal position of the electrode was identified when the firing of the neuron was robustly modulated by the positive current injection. Modulation was performed for at least 5 min to obtain reliable labeling. In the second protocol, for VTA DA neurons, after the extracellular recordings were made, using a 35–50-ΩM electrode, AC pulses were given in order to gain intracellular access. The amount of current in each AC pulse was progressively increased, and the electrode moved closer in 2 μm steps until the intracellular medium was accessed (evidenced by spike waveform change from biphasic to monophasic and a small negative shift in potential measured by the electrode; 5 to 25 mV). Then, if necessary, the electrode was moved up to 2 μm closer to the cell, and the recording was stabilized by the negative current. For labeling, a micro square current was applied (0.4–1 nA positive current, 200 ms duration, 50% duty cycle) for 10–20 min. In both protocols, after the current injection, the neurobiotin was left to transport along neuronal processes for at least 2 h. After the labeling sessions, the animals were perfuse-fixed with 25 ml of PBS, pH 7.4, followed by 50 ml 4% paraformaldehyde (w/v) in phosphate buffer, pH 7.4. Finally, the brains were post-fixed in 4% paraformaldehyde in PBS overnight, maintained in 30% sucrose in distilled water for 48 h, and sectioned.

### Electrophysiological Analysis

Electrophysiological analysis was carried out for the 25 (12 SNc and 13 VTA, see above) DA neurons that were recorded under urethane anesthesia. The other five neurons (three SNc and two VTA) were not analyzed as they came from a pool of neurons previously characterized using an alternative anesthetic regime (Farassat et al., 2019), which made direct electrophysiological comparisons difficult. Measurements of spike firing rate (FR), coefficient of variation (CV), coefficient of variation 2 (CV2), and percentage of spikes in bursts (% SIB) were taken from 3–15 min of spontaneous activity before the labeling session. Following spontaneous activity and before labeling, a strong somatosensory stimulus challenge was applied to the hind paw, the analysis of which will be reported elsewhere. To determine FR, the spontaneous activity train was binned into 0.5 s bins, and frequency was determined for each bin and averaged. CV was calculated as the standard deviation divided by the mean of the inter-spike intervals (ISI). CV2 was calculated for every single spike in the time series. As such, the standard deviation of the two adjacent ISIs was calculated and then divided by their mean and finally multiplied by √2. The overall reported CV2 is the average of every spike CV2 (Holt et al., 1996). The percentage of spikes in burst (% SIB) was determined from a suitable open access script developed by CED to interface with Spike2^1^. Bursts were composed of at least three spikes, and the classical criteria outlined by Grace and Bunney (1984) were used: a burst begins when two action potentials occur within 80 ms of each other and ends when an ISI greater than 160 ms occurs.

### Neuronal Identification

The brains were cut in the coronal plane on a freezing-stage microtome (Reichert–Jung Hn-40) at 25 or 40 μm. All midbrain-containing sections were incubated with Cy3-conjugated streptavidin (1:1,000; Jackson ImmunoResearch, USA) for 2–3 h to reveal the neurobiotin. After mounting, the sections were examined with an epifluorescent microscope (Nikon Eclipse Ci; Nikon, Japan) to confirm that the neurons were completely filled with tracer. One or two sections were selected, blocked with 3% normal horse serum (NHS) in PBS (v/v; Jackson ImmunoResearch), and incubated with a guinea pig anti-tyrosine hydroxylase (TH) antibody (1:1,000; Synaptic Systems, Germany) in PBS, 3% NHS, and.3% Triton-X overnight at room temperature. They were then incubated in Alexa Fluor 488 or Dylight 405-conjugated donkey anti-guinea pig antibody (1:1,000; Jackson ImmunoResearch). Three to four 8-min washes were performed in between and after incubation in streptavidin or antibodies. Labeling for neurobiotin and colocalization of neurobiotin-labeled processes with TH was assessed. Only neurons that were neurochemically identified as DA neurons by immunoreactivity for TH were analyzed further. An almost identical protocol was used for SNc DA neuron identification [see Meza et al. (2018) for further details]. Three SNc and two VTA DA neurons were identified as previously described in Farassat et al. (2019).

### Microscopy and Imaging

For the 13 VTA and 12 SNc DA neurons, fluorescence imaging for all neurobiotin-labeled profiles across sections was performed with one of the following three laser-scanning confocal microscopes: Nikon Eclipse C2, using the NIS-Elements C program (Nikon software) to acquire and export images (12 SNc and 3 VTA neurons), Zeiss LSM 700, using the ZEN2012 program (Zeiss software) (2 VTA neurons), and Olympus FV1000 using the Fluoview program (Olympus software) (7 VTA neurons). Low-magnification images were acquired with appropriate 10× and 20× objectives. High-magnification and z-stack images were acquired with a 60× oil or water immersion objective (1.3–1.4 numerical aperture). Images taken for 3D neuronal reconstruction were 512 pixels × 512 pixels in size with a resolution of 0.19 (2 VTA neurons) or 0.41 μm/pixel (12 SNc and 11 VTA neurons) and taken in z-stacks of 0.5 μm steps between images. To ensure the best signal-to-noise ratio in all the stack images, maximum and minimum intensity pixels were established independently in each channel and for each z-stack acquired during the acquisition sessions using the appropriate software [see also Meza et al. (2018)]. For the three SNc and two VTA DA neurons, high magnification z-stacks of juxtacellularly labeled neurons were acquired with a 60× oil immersion objective (1.4 numerical aperture) using a laser-scanning microscope (Nikon Eclipse90i, Nikon GmbH) and the NIS-Elements C program (Nikon software) with a 1,024 pixel × 1,024 pixel size [for further details see Farassat et al. (2019)].

### Neuronal Reconstructions

The dendritic domain of neurons selected for digital reconstruction was completely filled, and all of the dendrites extended to natural tapering ends. The axon was traced as it branched off from a proximal dendrite or cell body (Meza et al., 2018), did not exhibit local collaterals, and was followed until it eventually joined the nigrostriatal or medial forebrain bundle pathways, where its signal usually started to fade out. Neurons were reconstructed in three dimensions from all the z-stack images taken with the confocal microscope using Neurolucida (MBF Bioscience, USA). Neuronal fragments from every section were traced onto a corresponding digital section using the Serial Section Manager in Neurolucida (Henny et al., 2014). For the entire somatodendritic domain, a correction factor in the z-axis was applied to account for the shrinkage that follows dehydration and histological processing, which, in 25 cases, was approximately 50% and in five cases (from Farassat et al., 2019) was 34%. Quantitative data for anatomical parameters were obtained using the Neurolucida Explorer software.

### Morphological Analyses

To determine the location of each reconstructed SNc and VTA DA neuron, double immunostaining for TH and neurobiotin was performed in the section containing the soma or dendrite. The SNc and VTA were delimited in the TH-stained cell body section and compared with the sections provided by Franklin and Paxinos (2007) and Fu et al. (2012). Then, a virtual 3D map of the substantia nigra [SN, including pars compacta (SNc), pars lateralis (SNl), and pars reticulata (SNr)], all VTA constituent nuclei [including rostral VTA (VTAR), parabrachial pigmented (PBP), paraintrafascicular (PIF), paranigral (PN), infrascapular (IF), and caudal (CLi) and rostral (RLi) linearis], and relevant landmark tracts, was created in Neurolucida based on a recent study that re-assessed the boundaries and cyto-architecture of DA cluster groups (Fu et al., 2012). We decided to aggregate, in a single subdivision (SNc), all the SNc clusters defined by Fu et al. (2012), except the SNl, which we left apart as a different nucleus. Individual neuronal reconstructions were placed in the 3D map according to the ML, DV, and AP locations of the cell body obtained from the TH immunostaining. The neuronal reconstructions were further tilted-corrected by matching the position of the furthest dendritic tips to anatomical landmarks, to the best of our possibilities. Reconstructions of neurons labeled in the left hemisphere were vertically flipped and projected onto the right hemisphere. To predict the proportion of dendrites in a given subdivision, the entire neuronal reconstruction and 3D map were observed in the 3D module of Neurolucida, and rotated until the approximate location of a dendrite as it coursed through an adjacent subdivision was determined. Dendrites were detached from the main reconstruction and analyzed separately. Physical and topological data of dendrites and entire dendritic domains were taken from the Neurolucida Explorer software.

### Statistical Analysis

To assess whether data sets were normally distributed, we performed single-sample Kolmogorov–Smirnov test or, if the n was too small, Shapiro–Wilk normality test in all the data sets. For parametric data, unpaired *t*-test was performed (Figure 4). Non-parametric tests were performed on non-normally distributed data; two-tailed Mann–Whitney U tests (Figure 4) and Spearman correlation were performed (Figures 2, 4–7 and Supplementary Tables 1, 3–5). Significance for all the statistical tests was set at *p* < 0.05. Boxplots are explained in Figure 4 legend. All the statistical analyses were performed using GraphPad Prism 7.

## Results

**FIGURE 1:**
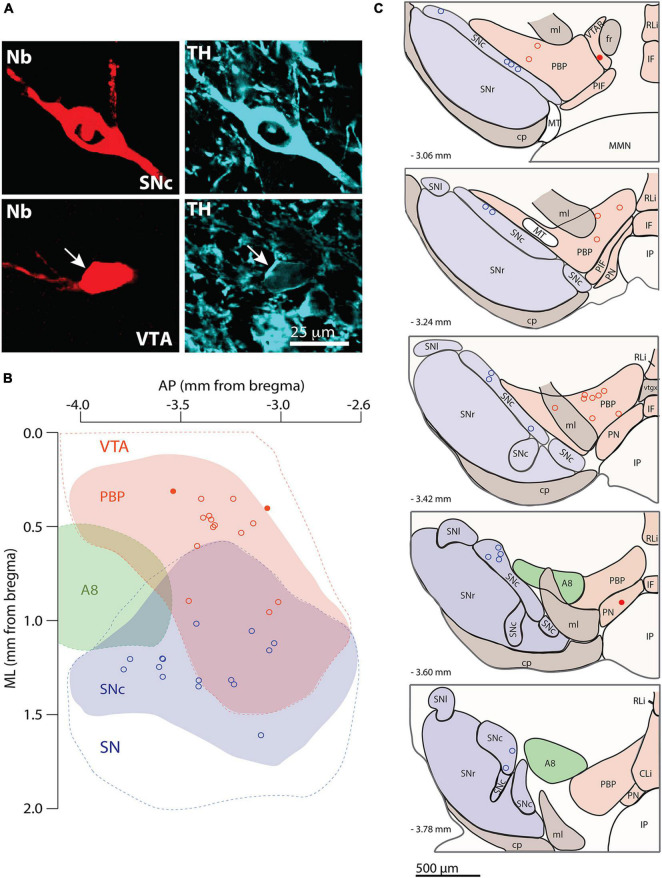
Labeling, identification, and cell body localization of dopaminergic (DA) neurons. **(A)** SNc (top) and VTA (bottom, arrow) neurons were labeled with neurobiotin (red, left) and expressed tyrosine hydroxylase immunoreactivity (cyan, right, arrow in bottom image). **(B,C)** Location of cell bodies of the 15 SNc and 15 VTA DA neurons used in this study in one **(B)** dorsal and five **(C)** frontal views. Blue, red, and green shades indicate the SNc, PBP, and A8 groups, respectively. Blue and red dashed lines indicate the substantia nigra and VTA regions, respectively in panel **(B)**. AP, anteroposterior; ML, mediolateral; CLi, caudal linear nucleus; cp, cerebral peduncle; fr, fasciculus retroflexus; IF, interfascicular nucleus; IP, interpeduncular nucleus; ml, medial lemniscus; MMN, medial mammillary nuclei; MT, medial terminal nucleus; PBP, parabrachial pigmented nucleus; PIF, parainterfascicular nucleus; PN, paranigral nucleus; RLi, rostral linear nucleus; SN, substantia nigra; SNc, substantia nigra pars compacta; SNl, substantia nigra compacta pars lateralis; SNr, substantia nigra pars reticulata; VTAR, rostral VTA.

Given the role of the dendritic domain in the afferent connectivity and activity of a neuron, two main questions guided this study. First: what are the architectural characteristics that define the dendritic domain of individual mouse SNc and VTA DA neurons? And, second: does the dendritic domain architecture relate to *in vivo* spontaneous firing activity at the individual cell level? To address these questions, we reconstructed the somatodendritic domain of DA neurons in 3D. We used the juxtacellular technique to label one neuron per hemisphere, either in the SNc or the VTA (Meza et al., 2018; Farassat et al., 2019) and subsequently identified them as DA by the expression of the catecholamine synthetic enzyme tyrosine hydroxylase (TH) (Figure 1A). We determined the ML, DV and AP locations of the cell bodies of reconstructed neurons in relation to the standard map of Fu et al. (2012). The cell bodies of labeled neurons located across the SNc (15 neurons) and VTA (15 neurons) (Figures 1B,C). In the case of SNc, cell bodies located at the central (in relation to mediolateral coordinates) and slightly posterior locations. In the case of the VTA, the cell bodies tended to locate at the more medial and anterior locations. In the VTA, 13 cell bodies located at various DV depths in the PBP (Figure 1C), and two others located in the PN and a VTAR, near the PBP (Figure 1C).

**FIGURE 2:**
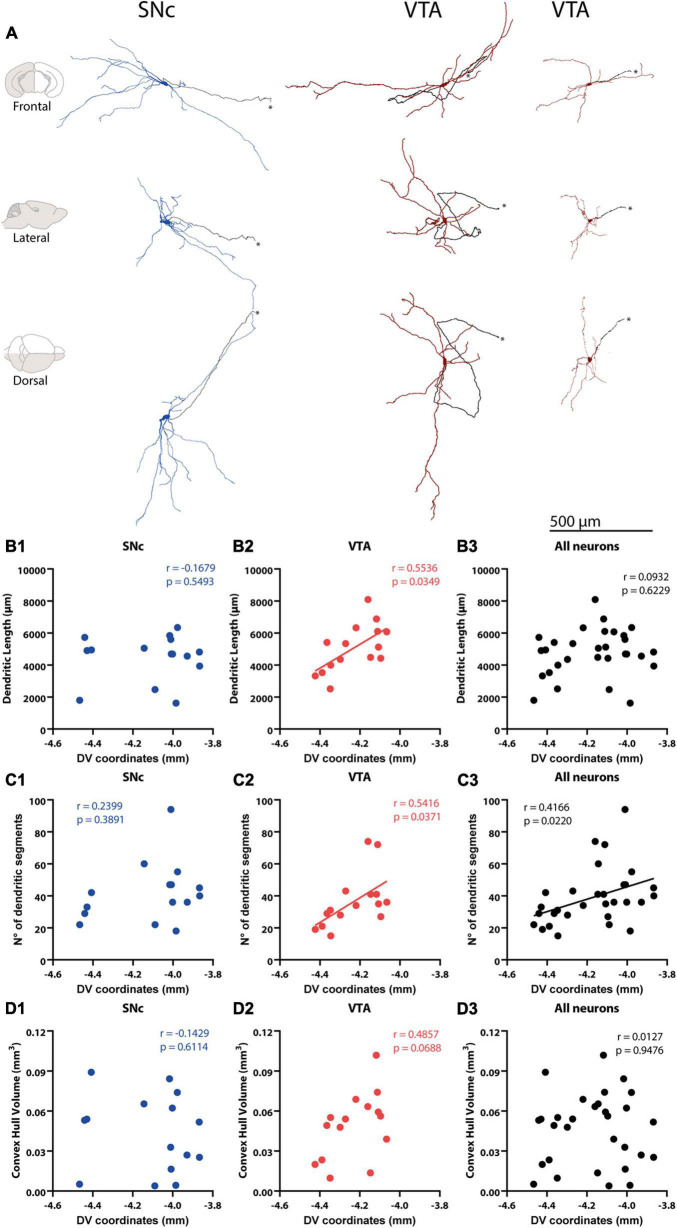
Reconstruction and correlation between morphological characteristics and cell body localization. **(A)** Identified SNc and VTA DA neuron 3D reconstructions. Arranged in columns, one SNc, and two VTA DA neurons are shown from the frontal (top), lateral (middle), and dorsal (bottom) views. Axons are shown in black in all panels and pinpointed with an *. Brain diagrams to the left indicate hemisphere depicted (in gray) and view. **(B1–D3)** Correlations between physical (dendritic length, convex hull) and topological (number of dendritic segments) measures and cell body position of 3D reconstructed SNc and VTA DA neurons. A positive correlation was found between total dendritic length and DV coordinates of VTA neurons **(B2)**, but this was not observed for SNc **(B1)** or all DA neurons pooled together **(B3)**. Number of dendritic segments and DV coordinates were positively correlated in VTA **(C2)**, and when all neurons were pooled together **(C1)** with no correlation observed for SNc DA neurons **(C2)**. No correlation was found between convex hull volume and DV position with all DA neurons **(D1)**, or for SNc **(D2)**, VTA **(D3)** for all DA neurons **(D1)**. Figure only shows correlations between morphology and DV position. Refer to the Results section and Supplementary Table 1 for correlations between morphology and mediolateral (ML) and anteroposterior (AP) axis positions.

### Physical and Topological Characteristics of Mouse Dopaminergic Neurons

We examined whether the architectural features of the dendritic tree differ between SNc and VTA DA neurons and, more generally, whether cell body position in these areas was a good predictor of dendritic tree architecture. To do that, the dendritic tree of labeled SNc and VTA DA neurons were reconstructed and analyzed by its physical and topological characteristics. For physical characteristics, we analyzed total dendritic length (the actual tortuous length, as taken from reconstructions) and convex hull volume [which corresponds to the polygon connecting the most distant dendritic tips or inflections of the dendritic domain of a neuron, as if a “plastic sheet (was) wrapped around the entire neuron”^2^], and used these characteristics as a proxy for volumetric maximal extension of the dendritic domain (Gertler et al., 2008; Vrieler et al., 2019). For topological characteristics, we analyzed number of primary trees, maximum dendritic branch order, and number of dendritic segments (the latter is closely related to number of dendritic nodes and ends). Quantitative analysis showed differences of up to 4 or 5 times in dendritic length and topological parameters between neurons, and over 10 times in convex hull volumes in both SNc (Meza et al., 2018) and VTA (Figure 2 and Table 1) populations. Regional comparisons showed that the SNc and VTA neurons did not differ in physical or topological characteristics (Table 1).

**TABLE 1:** Cell body and dendritic arbor size and complexity measures in ventral tegmental area (VTA) and substantia nigra pars compacta (SNc) reconstructed neurons.

Parameter	SNc (*n* = 15)			VTA (*n* = 15)		
	Average	SEM	Range	Average	SEM	Range
Dendritic Length (μm)	4,463	370	1,618–6,339	5,062	383	2,508–8,094
Soma Surface Area (μm^2^)	1,692	200	765–3,168	1,359	152	670–2,927
Convex Hull volume (mm^3^)	0.0432	0.0075	0.0038–0.0890	0.0490	0.0064	0.0097–0.1019
Dendritic Trees N°	5.4	0.43	2–8	4.90	0.36	2–7
Max. Dendritic order	6.4	0.63	3–13	6.08	0.50	3–9
Number of segments	41.7	4.86	18–94	36.50	4.37	15–74

*Depending on the distribution of the data-sets, an unpaired *t*-test or Mann–Whitney test was performed, and no significant differences were found between groups in the parameters tested.*

Because previous studies have reported that cell bodies of physiologically distinctive or projection-specific subpopulations aggregate at certain locations in the SNc or VTA (Lammel et al., 2008; Brischoux et al., 2009; Farassat et al., 2019), we wonder whether the dendritic morphology of neurons could also depend on cell body location within these regions. In the case of SNc neurons, we found that morphological characteristics did not relate to the position in the DV (Figures 2B,C1,D1), ML and AP axes (Supplementary Table 1). In the VTA, on the other hand, we found a positive correlation among DV position and dendritic length (Figure 2B2), number of dendritic segments (Figure 2C2), and maximum dendritic order (Supplementary Table 1), in that the dendritic tree of neurons whose cell bodies locate more dorsally shows longer dendrites that are arranged in a more complex manner. A positive relationship between DV position and maximum dendritic order (Supplementary Table 1) and number of segments (Figure 2C3) was also found when the SNc and VTA neurons were pooled together. Dendritic domain convex hull volumes did not correlate with cell body position for SNc, VTA, or the entire population. In summary, the results show that dendritic morphological diversity is related to cell body position in the VTA and entire population, in that the dendritic length and complexity of VTA neurons increase toward more dorsal positions. They also indicate that morphological diversity could be a factor underlying differences in electrophysiological profile across neurons within the VTA and substantia nigra regions.

### Course of Individual Neuron Dendritic Tree Across SNc and VTA Regions and Subdivisions

**FIGURE 3:**
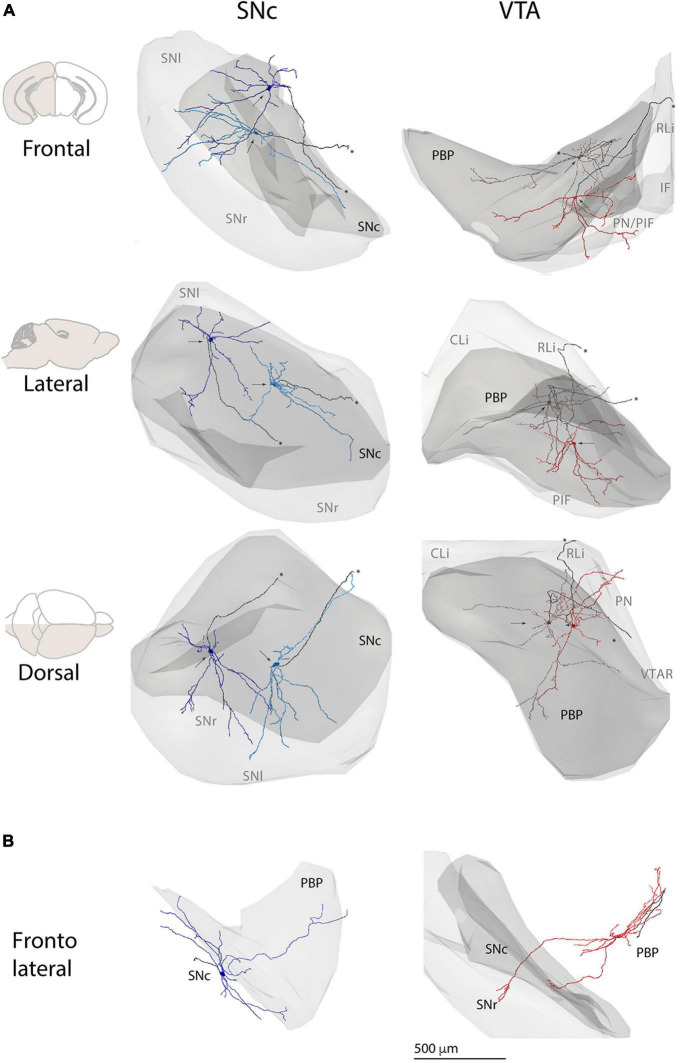
Cell body and dendritic domain of individual substantia nigra and VTA neurons within and between respective regions. **(A)** 3D reconstructions of two SNc (left column) and two VTA (right column) DA neurons and respective regions. Neurons were placed inside 3D renderings of substantia nigra and VTA, as part of a common reference map based on Fu et al. (2012). Depicted in light gray is the substantia nigra (left) or the VTA (right) and in darker gray is the SNc or PBP. Gray text indicates the projected location of SN or VTA subdivisions. Arrows indicate the position of cell bodies. Axons are in black and marked with an ^∗^. For SNc DA neurons, note the extension of dendrites outside the boundaries of SNc into the SNr (frontal and dorsal views). For the VTA DA neuron depicted in bright red, note the extension of dendrites outside the boundaries of the PBP, into more ventral VTA subdivisions PIF and PN (frontal and dorsal views). **(B)** 3D reconstructions of one SNc (left) and one VTA DA neurons (right) depicting dendritic extensions onto PBP and SN, respectively. Only PBP (left) and SNc-SNr (right) contours are shown. CLi, caudal Linear nucleus; IF, interfascicular nucleus; PBP, parabrachial pigmented nucleus; PIF, parainterfascicular nucleus; PN, paranigral nucleus; RLi, rostral linear nucleus; SN, substantia nigra; SNc, substantia nigra pars compacta; SNl, substantia nigra compacta pars lateralis; SNr, substantia nigra pars reticulata; VTAR, rostral VTA.

Because the substantia nigra and the VTA encompass several cytoarchitectonic subdivisions (Fu et al., 2012), that in the case of substantia nigra are well characterized to be neurochemically and hodologically distinctive (Bolam and Smith, 1990; Bolam et al., 1991; Comoli et al., 2003; Henny et al., 2012) we estimated the dendritic extension across different subdomains for individual neurons. To do that, we created a common 3D map of the substantia nigra, VTA, and respective subdivisions. Then we placed the reconstructions inside according to the location of their cell bodies (Figure 3) and in-tissue confirmed orientation of the dendritic tree (see section “Materials and Methods”). In general, most SNc neurons had dendrites descending into the SNr (Figure 3A), and some toward adjacent VTA (Figure 3B) and other nearby regions. In the case of PBP-located VTA neurons, the neurons extended dendrites into the underlying PIF and PN subdivisions (Figure 3A), and sometimes into the substantia nigra (Figure 3B). We quantified for each SNc neuron the proportion of dendrites in different nuclei and found that in the SNc neurons, an average of 39% of dendrites stayed in the SNc, over 33% in SNr, and the rest toward other adjacent regions, namely, dorsal tegmentum and adjacent lateral limb of the PBP (Supplementary Table 2). In the case of the VTA neurons, which in our sample included 13 neurons in the PBP and 2 in adjacent PN and VTAR, most dendrites located in the PBP (73%) and adjacent PIF and PN, or midline VTA nuclei (11%). We also observed occasional crossing-over to the adjacent substantia nigra (7%) (Supplementary Table 2). Given that subdivisions are characterized not only because of cytoarchitectural features of the resident neuronal somata (Fu et al., 2012) but also the specific pattern of afferent innervation and neurochemistry (Bolam and Smith, 1990; Bolam et al., 1991; Comoli et al., 2003; Henny et al., 2012), the results indicate the capacity of neurons to sample and integrate inputs arriving at differentiated loci. From a cellular point of view, these results also show that the dendritic tree of DA neurons can be seen as a multicompartmental domain, as it is the case for rat SNc neurons (Hajós and Greenfield, 1994; Henny et al., 2012) and may play a role in physiological diversity.

**FIGURE 4:**
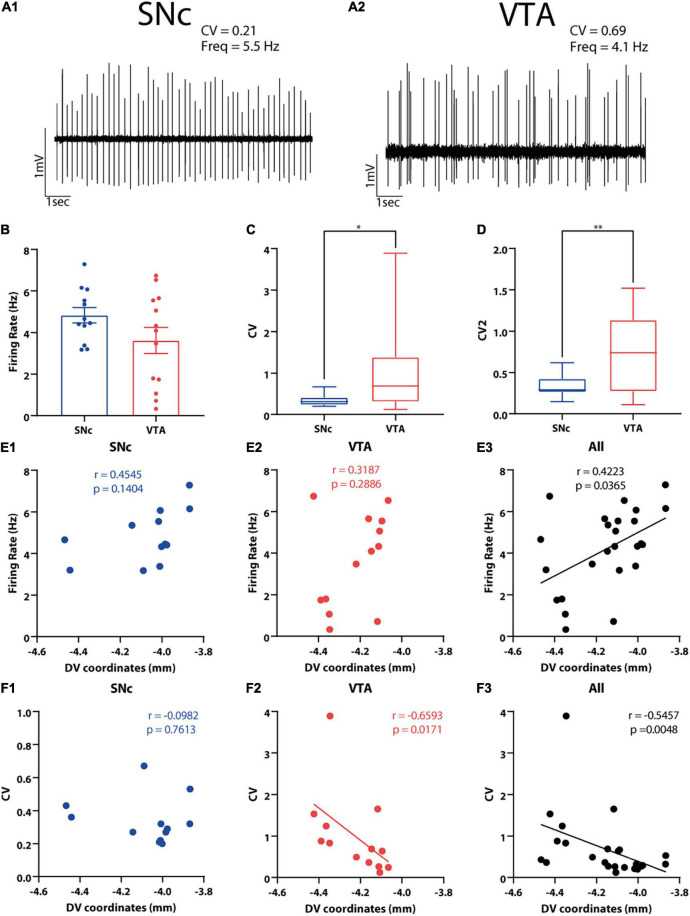
*In vivo* electrophysiological characteristics of SNc and VTA DA neurons and their correlation with cell body position in the DV axis. **(A1,A2)** Examples of *in vivo* extracellular recordings of **(A1)** SNc and **(A2)** VTA neurons. **(B–D)** Electrophysiological properties of SNc and VTA DA neurons were compared. Firing rate **(B)** and the firing regularity measures **(C)** CV and **(D)** CV2 are shown. ^∗^*p* < 0.05, ^∗∗^*p* < 0.01, unpaired *t*-test (CV2) or Mann–Whitney *U* test (CV). **(E1–F3)** Correlations between electrophysiological variables and DV cell body position of 3D-reconstructed SNc and VTA DA neurons. Positive correlation was found between firing rate and DV coordinates when all neurons were analyzed together **(E3)**, but this was not observed in each area separately **(E1,E2)**. Negative correlation was observed between CV and DV coordinates used and for VTA **(F2)** and for all neurons **(F3)** but not for SNc DA neurons **(F1)**. Figure only shows correlations between electrophysiological variables and DV position. Refer to the Results section and Supplementary Table 3 for correlations between electrophysiological variables and ML and AP axes position.

**FIGURE 5:**
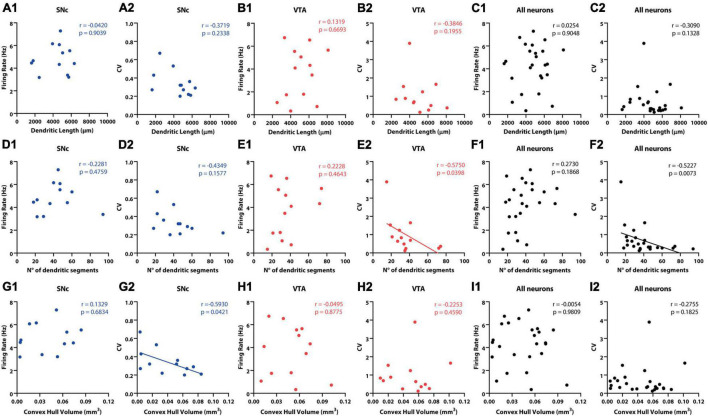
Correlation between *in vivo* electrophysiological and morphological characteristics of SNc and VTA DA neurons. **(A1,B1,C1)** No significant correlation between dendritic length and firing rate was found for **(A1)** SNc, **(B1)** VTA, or all **(C1)** DA neurons. **(A2,B2,C2)**. No significant correlations were found between CV and dendritic length for **(A2)** SNc, **(B2)** VTA, or all **(C2)** DA neurons. No significant correlation between number of dendritic segments and firing rate was observed for **(D1)** SNc, **(E1)** VTA, or all **(F1)** DA neurons. **(D2)** No correlation was found between CV and number of dendritic segments for SNc neurons. On the other hand, a negative correlation was found for **(E2)** VTA and for **(F2)** all neurons. Finally, no correlation between convex hull volume and firing rate was found for **(G1)** SNc, **(H1)** VTA, or all **(I1)** DA neurons. Negative correlation was found between convex hull volume and CV in **(G2)** SNc neurons, but not in panel **(H2)** VTA or all **(I2)** DA neurons. See Results and Supplementary Tables for further physiological and anatomical correlations.

**FIGURE 6:**
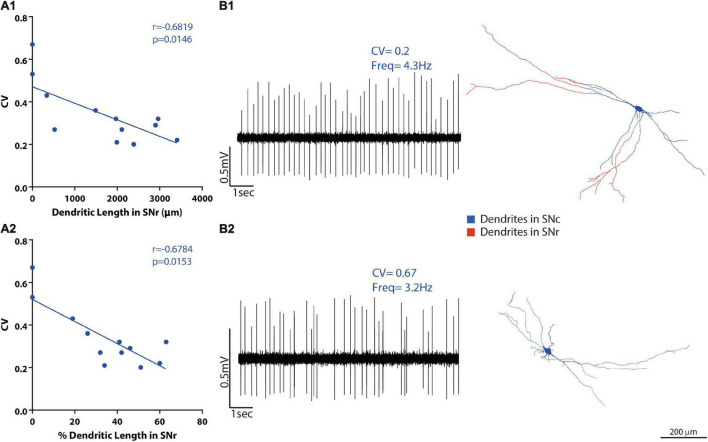
SNc DA neuron heterotopic distribution of dendrites correlates with the electrophysiological activity of the neurons. **(A1-2)** Firing regularity (coefficient of variation, CV) correlations with the proportion of dendritic length in panel **(A1)** SNr, but not in panel **(A2)** SNc. **(B1-2)** Raw data examples of firing traces of SNc DA neurons and their matching 3D reconstructions. Examples of one [**(B1)**-top] regular and one [**(B2)**-bottom] irregular SNc raw baseline recordings (10 s) with their respective dendritic reconstructions [**(B1,B2)**, right] showing dendrites inside SNr in red and their dendrites inside SNc (and other subdivisions, not shown) in blue. Scale bar = 200 μm.

**FIGURE 7:**
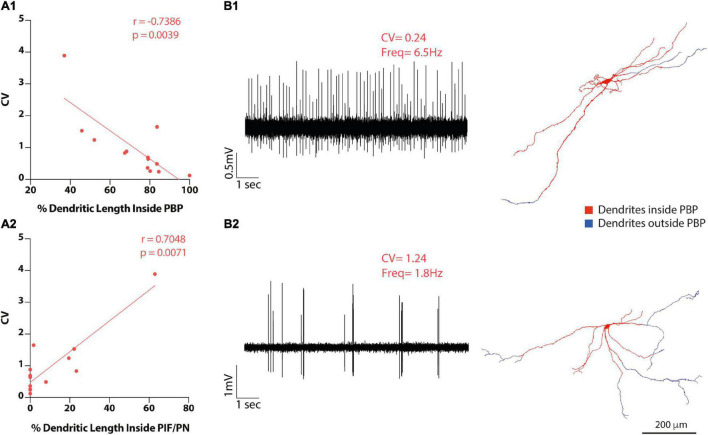
VTA DA neuron heterotopic distribution of dendrites correlates with baseline electrophysiological activity. **(A1-2)** Firing regularity (CV) correlations with the proportion of dendritic length inside PBP **(A1)** or inside PIF/PN subdivisions. **(B1-2)** Raw data examples of firing traces of VTA DA neurons and their matching 3D reconstructions. Examples of one [**(B1)**-top] regular and one [**(B2)**-bottom] irregular VTA DA neuron raw baseline recordings (10 s) with their respective dendritic reconstructions [**(B1,B2)**, right] showing dendrites inside PBP in red and their dendrites outside PBP in blue. Neuron in B2 is also shown in Figure 3. Scale bar = 200 μm.

### Cell Body Position and Spontaneous *in vivo* Activity

In order to examine the relationship between single cell dendritic morphology and spontaneous activity, we analyzed the firing behavior of 25 out of the 30 neurons (12 from SNc and 13 from VTA, see section “Materials and Methods”). We chose these 25 neurons because they all had been recorded under the same anesthetic regime (urethane). We found that spontaneous firing rate did not differ between the SNc and VTA neurons [*t*(23) = 1.625, *p* = 0.1177 unpaired *t*-test, Figures 4A,B], which could in part be explained by the large firing rate variability in the VTA group (Figure 4B). On the other hand, we found that the SNc neurons fired significantly more regularly than the VTA neurons, as evidenced by lower CV and CV2 values [CV: *U* = 36.5, *p* = 0.028 Mann–Whitney test; CV: *t*(23) = 2.84, *p* = 0.0093 unpaired *t*-test, Figures 4A,C]. We did not find a significant difference between groups at the level of bursting activity, computed as the percentage of spikes in burst (SFB) [*t*(23) = 1.629, *p* = 0.117 unpaired *t*-test]. We did notice, however, that the number of neurons showing at least one burst event was larger in the VTA sample (10 out of 13 neurons) than in the SNc sample (6 out of 12). We noticed that the overall incidence of bursting activity in neurons appeared low, which, in part, could be explained by the use of urethane that, as shown for SNc neurons, reduces bursting activity (Tepper et al., 1995).

We checked whether cell body localization correlated with electrophysiological characteristics (Figures 4E,F and Supplementary Table 3). We found that firing rate increased toward dorsal positions when we pooled both the SNc and VTA neurons together (*r* = 0.4223, *p* = 0.0365 Spearman correlation, Figure 4E3), although this relation was lost when observed in each region separately (SNc: *r* = 0.4545, *p* = 0.1404, VTA: *r* = 0.3187, *p* = 0.2286 Spearman correlation, Figures 4E1,E2 and Supplementary Table 3). We also found that firing irregularity increased toward more ventral positions when all the neurons were pooled together, reflected in larger CV at deeper DV positions (*r* = −0.5457, *p* = 0.0048 Spearman correlation, Figure 4F3). That relationship was maintained for the VTA neurons alone (*r* = −0.6593, *p* = 0.0171 Spearman correlation, Figure 4F2 and Supplementary Table 3) although not for the SNc neuron (*r* = −0.0982, *p* = 0.7613 Spearman correlation, Figure 4F1 and Supplementary Table 3) values. Firing activity was also studied in relation to location of the cell body in the ML and AP axes. When considering all neurons, a negative relationship between the ML position and CV (also CV2) values was found (CV: *r* = 0.4491, *p* = 0.0243 Spearman; CV2: *r* = 0.4047, *p* = 0.0448 Spearman correlation, Supplementary Table 3), in that more lateral neurons fired more regularly, reflecting the already mentioned significant difference in regularity between the SNc and VTA neurons (Figures 4C,D). We also found that the neurons fired more irregularly at posterior positions (CV2: *r* = 0.4359, *p* = 0.0294 Spearman correlation, Supplementary Table 3). Finally, we observed that more anterior localization was associated to faster firing in the case of SNc neurons (*r* = −0.6993, *p* = 0.0145 Spearman correlation, Supplementary Table 3) and more bursting activity in the case of VTA neurons (SIB: *r* = 0.6630, *p* = 0.0135 Spearman correlation, Supplementary Table 3). In summary, cell body position was a good, although complex, predictor of spontaneous firing behavior in DA neurons.

### Dendritic Domain Morphology as a Correlate of *in vivo* Activity

Given that cell body localization was a good predictor of spontaneous activity (Figure 4) and that cell body position itself is also associated to differences in physical and topological characteristics of dendritic trees (Figure 2), we examined how these morphological and electrophysiological characteristics are related to each other (Figure 5 and Supplementary Table 4). In the case of SNc neurons, no relationship between electrophysiological (firing rate, CV, CV2 or SIB) and morphological (dendritic length, number of dendritic segments, maximum branch order) variables was found (Figure 5 and Supplementary Table 4). On the other hand, however, we found that larger dendritic volumetric space (larger convex hull volumes) values correlated positively with firing regularity (CV: *r* = −0.5930, *p* = 0.0421 Spearman correlation, Figure 5), although not with firing rate or SIB (firing rate: *r* = 0.1329, *p* = 0.6834; SIB: *r* = −0.4703, *p* = 0.1229 Spearman correlation, Figure 5 and Supplementary Table 4). In the case of VTA neurons, firing rate, CV2, or SIB did not relate to morphological variables (Figures 5C2,D2 and Supplementary Table 4). However, CV values in VTA neurons negatively correlated to number of dendritic segments (*r* = −0.575, *p* = 0.0398 Spearman correlation, Figure 5 and Supplementary Table 4), in that a more complex architecture relates to more regular firing. We did not find that the convex hull volume of VTA neurons related to any electrophysiological variable (Figure 5 and Supplementary Table 4). Finally, when the entire population was considered, we found that neither firing rate nor SIB (Supplementary Table 4) related to dendritic length, maximum dendritic order, number of segments, or convex hull volume (Supplementary Table 4). However, the CV and CV2 values related to maximum dendritic order and number of segments (Supplementary Table 4). In summary, morphological features of the dendritic domain relate to electrophysiological characteristics in individual DA neurons in that a more extensive dendritic space (in the case of SNc neurons) or a more complex architecture (in the case of VTA neurons or the entire population), leads to more regular firing.

### Dendritic Compartmental Organization as a Correlate of *in vivo* Activity

Having found that dendritic tree organization relates to *in vivo* spontaneous activity diversity (above), and that an important property of the dendritic organization is the presence of dendrites across multiple SN or VTA subdivisions (Figure 3 and Supplementary Table 2), we examined whether the presence of dendrites across different SN or VTA subdivisions could also relate to spontaneous activity in individual neurons. For that, we tested whether absolute or proportional dendritic length in different subdivisions (Supplementary Table 2) correlated with basal electrophysiological parameters (Figures 6, 7 and Supplementary Table 5). In SNc neurons, the presence of dendrites in the SNr subdivision did not correlate with firing rate or SIB (Supplementary Table 5). On the other hand, baseline firing regularity values (CV and CV2) were negatively correlated with dendritic length (CV: *r* = −0.6819, *p* = 0.0146; CV2: *r* = −0.718, *p* = 0.0085 Spearman correlation, Figure 6A1) and dendritic length percentage (CV: *r* = −0.6784, *p* = 0.0153; CV2: *r* = −0.7321, *p* = 0.0068, Figure 6A2) in SNr (Supplementary Table 5). Hence, neurons with more dendrites in SNr fired more regularly (Figure 6B1), and neurons with little or no dendrites in SNr were more irregular (Figure 6B2). No relationship was found between the extension of dendrites in SNc, PBP, or tegmentum above SNc and electrophysiological parameters (Supplementary Table 5). Neurons also presented dendrites in the retrorubral field (*n* = 5) and in SNl (*n* = 1) but were few, and the dendritic length was proportionally very small; thus, we did not evaluate correlations regarding dendrites in these areas.

In VTA neurons, total dendritic length (or percentage) inside or outside the PBP subdivision did not correlate with firing rate or SIB either (Supplementary Table 5). On the other hand, firing regularity did relate to dendritic distribution in different subdivisions. Specifically, we found a negative correlation between absolute dendritic length (or dendritic length percentage) in the PBP and CV or CV2 values, in that neurons with a larger percentage of dendrites located in PBP fired more regularly (CV: *r* = −0.7386, *p* = 0.0039; CV2: *r* = −0.7165, *p* = 0.0059 Spearman correlation, Figures 7A1–B2 and Supplementary Table 5), and that those with a lower proportion of dendrites in PBP fired more irregularly (Figure 7B2). Several VTA DA neurons also had dendrites in the more ventrally located PIF and PN subdivisions of the VTA, and we found a positive correlation with either dendritic length or length percentage in PIF/PN and CV or CV2 values (% dendritic length, CV: *r* = 0.7048, *p* = 0.0071; CV2: *r* = 0.663, *p* = 0.0135 Spearman correlation, Figure 7A2 and Supplementary Table 5). Additionally, some neurons had dendrites in the tegmentum dorsal to the VTA (mRF/p1RF); however, there were no significant correlations of CV or CV2 with the total dendritic length (or the percentage (Supplementary Table 5). Very few neurons had dendrites in the A8 field (*n* = 1), VTAR (*n* = 4), ml (*n* = 1), thus, their dendritic extension in those areas was not evaluated for correlation with baseline electrophysiological measurements.

## Discussion

Mouse DA neuron dendritic domains show a considerable architectural diversity. Some of that diversity is explained by a dorsoventral gradient of further smaller and simpler dendritic domains at ventral positions, particularly evident in the VTA. Single cell dendritic architecture also predicts differences in spontaneous firing patterns. In the SNc, firing irregularity relates to smaller dendritic space, as quantified by convex hull volumes, and a smaller proportion of SNr-projecting dendrites. In the VTA, instead, irregularity is associated to cell body ventral localization, topologically simpler dendritic domains, and a smaller proportion of dendrites within the PBP.

### Morphology of Mouse SNc and VTA Dopaminergic Neurons

This study conforms to classical descriptions of single cell dendritic morphology in the rat SNc, characterized by extensive dendritic fields, relatively simple bifurcation patterns, and contingents of SNr-projecting dendrites (Juraska et al., 1977; Grace and Bunney, 1983; Tepper et al., 1987). It also follows descriptions of identified DA (Grace and Onn, 1989) or Golgi-stained VTA neurons (Phillipson, 1979) in rats that report extensive, overlapping, and radially oriented dendrites. In this study, we quantitatively analyzed single cell dendritic domains and found a considerable architectural diversity.

We did not find that diverse dendritic architectures of SNc neurons associated with the DV, ML, or AP cell body position. In contrast, VTA DA neurons, being similarly diverse, exhibited a dorsoventral gradient of reduced size and complexity of dendritic domains, in line with the study of Philipson showing ventral PN neurons that appeared smaller than dorsal PBP ones (Phillipson, 1979). We acknowledge that our sampling may fall short of revealing subtler subregional differences in morphology. Future studies that will perform more systematic and extensive sampling, as carried out in other brain areas (Benavides-Piccione et al., 2006; Vrieler et al., 2019), may provide beneficial in this respect.

We wondered whether single cell morphological diversity could also relate to the course of dendrites within substantia nigra and VTA cytoarchitectonic subdivisions. We confirmed that a proportion of SNc neurons project dendrites to the SNr. Similar to what we described in the rat (Henny et al., 2012), we failed to observe a clear association between ventral or dorsal tiers cell body location with exhibiting, or not, SNr-projecting dendrites, as previously reported (Gerfen et al., 1987; Grace and Onn, 1989). In fact, the cell bodies of most SNc neurons in this study, such as those exhibiting SNr-projecting dendrites, locate in what would correspond to the neurochemically and hodologically defined mouse SNc dorsal tier (Fu et al., 2012; Figure 1). We also observed (Figures 2, 3) that SNr-projecting dendrites usually originate from multiple primary dendrites and course across the SNr in various directions, and that they do not from a single thick *apical* dendrite, as sometimes reported (Juraska et al., 1977; Gerfen et al., 1987; Yung et al., 1991). As also evident from the long dendritic extension of VTA neurons (Figure 3), we show that dendrites course across subdivisions of the VTA. The functional consequences of such heterotopic distribution of dendrites should be interpreted in relation to the segregated distribution of input into different subdivisions, as demonstrated for SNc versus SNr (Gerfen et al., 1985; Bolam and Smith, 1990; Bolam et al., 1991). This is a much more difficult challenge for VTA neurons, for the segregation of inputs to different subdivision of the VTA seems to be minimal, as described in rats (Geisler and Zahm, 2005).

### Activity Correlates of Neuron Localization and Dendritic Domain Architecture

We described a dorsoventral gradient of increased firing irregularity for VTA (and SNc-VTA) DA neurons. Interestingly, a recent study has also reported an effect of DV cell body positioning in *in vivo* firing frequency of identified mouse VTA DA neurons, in that lateral nucleus accumbens-projecting neurons (which locate dorsally in the VTA and include medial SNc neurons) were faster than medial nucleus accumbens-projecting neurons (located ventrally in the VTA) (Farassat et al., 2019). We also found that anterior cell body locations associated with more bursting activity in the VTA, but failed to find differences in mediolateral positioning, contrasting with evidence that dorsolateral striatum (DLS)-projecting lateral SNc neurons are burstier than DLS-projecting medial SNc neurons (Farassat et al., 2019). Although these differences may, in part, be due to the smaller sample in this study or anesthetic regime, it is also likely that other anatomical principles underlie functional diversity beyond cell body position, and may include projection target (Farassat et al., 2019), axon initial segment size (González-Cabrera et al., 2017; Meza et al., 2018), size and organization of somatodendritic or proximal dendritic domains (Jang et al., 2014; Meza et al., 2018; Moubarak et al., 2019) or, as shown in this study, the organization of the dendritic domain.

In fact, regularity of firing in SNc neurons is correlated with dendritic extension, as measured by convex hulls. One could assume that total dendritic length, which may affect convex hull size, could also relate to regularity, but it does not. Regularity of discharge, instead, was associated specifically with the proportion of SNr-projecting dendrites, indicating that it is not dendritic extension *per se* that underlies regularity but specific properties of SNr-projecting dendrites. A crucial difference between SNr-projecting versus SNc dendrites is the denser GABAergic input in the former (Henny et al., 2012). It has been previously recognized for cortical integrate-fire neurons that firing regularity is tuned by changes in synaptic excitation/inhibition (E/I) balance (Hamaguchi et al., 2011). Therefore, an enhanced inhibitory tone onto SNr-projecting dendrites might promote regularity, an interpretation consistent with the role of inhibition in suppressing excitation-mediated firing irregularity and bursting activity (Tepper et al., 1995; Celada et al., 1999). In addition, enhanced inhibition might boost the role of subthreshold conductances such as T-type calcium channels and coupled Ca2 + activated SK channels, which themselves further promote regular discharge (Wolfart and Roeper, 2002). This is not to say, however, that inhibition should always promote regularity and emergence of regular pacemaking firing in DA neuronal subtypes. Indeed, a recent report showed that some DA neurons may exhibit *in vivo* hyperpolarization-initiated rebound bursting (Otomo et al., 2020) in line with previous *ex vivo* evidence showing that rebound excitation may also depend on T-type channels in calbindin-negative SNc DA neurons (Evans et al., 2017; Gantz et al., 2018).

In VTA DA neurons, regularity was predicted by the dorsal positioning of the cell body, and dendritic domain complexity and extension within the PBP. Again, it is noticeable that regularity was not related to dendritic size *per se*, but tree complexity and heterotopic distribution. As mentioned earlier, evidence shows that, at least when considering the entire population of inputs to the VTA, they do not seem to segregate according to subdivisions (Geisler and Zahm, 2005) (their Figure 14). One plausible, although highly speculative, hypothesis that could explain a regional effect on firing regularity may be that somata and dendrites located in the PBP receive a high inhibitory-to-excitatory innervation ratio from local or extrinsic afferents (Omelchenko and Sesack, 2009; Faget et al., 2016), therefore mimicking the high inhibitory-to-excitatory innervation ratio seen in SNr-projecting dendrites.

### Functional Compartmentalization in Proximal and Distal Dendritic Domains

Previous studies have shown the role that dendrites play in synaptically mediated phenomena such as burst firing (Wilson and Callaway, 2000; Komendantov et al., 2004; Blythe et al., 2007; Gantz et al., 2018; Lopez-Jury et al., 2018) and firing inhibition or pauses (Hajós and Greenfield, 1994; Henny et al., 2012; Paladini and Roeper, 2014). Our data support this role and suggest that dendritic organization influences firing pattern by allowing irregular and burst firing to emerge (which we assume reflects excitatory volleys of activity reaching the dendritic domain), or, following afferent inhibitory activity, to enhance regularity or rebound bursting (Otomo et al., 2020).

The influence of the dendritic domain on firing pattern, on the other hand, sharply contrasts with the null correlation between dendritic domain (size, complexity, extension, heterotopic distribution) and firing frequency (Figure 5 and Supplementary Tables 4, 5). This supports the contention that DA neurons could be seen as functionally compartmentalized structures with different degrees of influence on firing pattern (e.g., its dendritic domain) or firing frequency [e.g., its more proximal subcellular compartments such as cell body, proximal dendrites (Jang et al., 2014), and axon initial segment (Meza et al., 2018)]. In fact, we have shown that axon initial segment size strongly correlates with *in vivo* spontaneous firing frequency but not with irregular or burst firing (Meza et al., 2018). In SNc neurons, compartmentalization should also extend to functionally and synaptically differentiated dendrites that locate either in the SNc or SNr. Due to a strong GABAergic influence, SNr dendrites could promote regular firing (see above) while also facilitating the concerted action of inhibitory inputs during aversive stimulation (Henny et al., 2012; Paladini and Roeper, 2014). Conversely, due to a higher proportion of excitatory glutamatergic and cholinergic synapses on SNc dendrites (Bolam et al., 1991; Henny et al., 2012), they could be better suited to mediate phasic bursting and/or irregular firing. Regarding the VTA, recent evidence has reported differences in excitability between axon-bearing and non-axon bearing dendrites (Engel and Seutin, 2015), supporting a compartmentalized view of VTA neurons proximal dendritic domain. Future studies that will describe differences in afferent innervation of VTA subdivisions or the distribution of inputs in the somatodendritic domain of individual neurons may shed light onto this subject. Finally, approaches aimed to test the role of different compartments in firing pattern, such as those that use subcellular specific expression of channel rhodopsins (Greenberg et al., 2011; Baker et al., 2016; Mahn et al., 2018), which could be coupled to focal illumination of opsins (Sun et al., 2014; Stahlberg et al., 2019), would help to test the causal relationship between morphological characteristics of different compartments of DA neurons and firing properties of the cell.

## Data Availability Statement

The raw data supporting the conclusions of this article will be made available by the authors, without undue reservation.

## Ethics Statement

The animal study was reviewed and approved by Ethics Committees of the School of Medicine of the Pontificia Universidad Católica de Chile and German Regierungspraesidium Darmstadt.

## Author Contributions

TM, NF, RM, and CG-C performed the experiments. TM, RG, NF, and PH contributed to the data analysis. TM, RG, JR, and PH contributed to the manuscript writing. All authors approved the submitted version.

## Conflict of Interest

The authors declare that the research was conducted in the absence of any commercial or financial relationships that could be construed as a potential conflict of interest.

## Publisher’s Note

All claims expressed in this article are solely those of the authors and do not necessarily represent those of their affiliated organizations, or those of the publisher, the editors and the reviewers. Any product that may be evaluated in this article, or claim that may be made by its manufacturer, is not guaranteed or endorsed by the publisher.

## Footnotes

1
http://ced.co.uk/downloads

2
https://www.mbfbioscience.com/help/nx11/Content/Analyses/Convex_Hull_Analysis.htm
