# Supplementary material for: Dendritic Architecture Predicts in vivo Firing Pattern in Mouse Ventral Tegmental Area and Substantia Nigra Dopaminergic Neurons
Source: Front Neural Circuits. 2021 Nov 19;15:769342. doi: 10.3389/fncir.2021.769342 (PMC8640462; doi:10.3389/fncir.2021.769342)
Supplement: Supplementary file 1 [file Table_1.docx]

Supplementary Material

| **Supplementary Table 1: Spearman correlation values between morphology and cell body position parameters of DA mesencephalic neurons** | | | | | | |
| --- | --- | --- | --- | --- | --- | --- |
|  | **SNc (n=15)** | | | | | |
|  | **ML** | | **DV** | | **AP** | |
|  | r | p | r | p | r | p |
| **Dendritic Length (µm)** | -0.0036 | 0.9949 | -0.1679 | 0.5493 | 0.2929 | 0.2887 |
| **Convex Hull Volume (µm^3^)** | 0.2071 | 0.4578 | -0.1429 | 0.6114 | 0.2857 | 0.3011 |
| **Nº Dendritic Trees** | 0.2345 | 0.4002 | 0.1832 | 0.5134 | -0.1979 | 0.4796 |
| **Maximum Dendritic Order** | -0.5128 | 0.0506 | 0.2111 | 0.4502 | 0.0370 | 0.8958 |
| **Nº of Dendritic Segments** | -0.2739 | 0.3231 | 0.2399 | 0.3891 | -0.1987 | 0.4776 |
|  | **VTA (n=15)** | | | | | |
|  | **ML** | | **DV** | | **AP** | |
|  | r | p | r | p | r | p |
| **Dendritic Length (µm)** | -0.2964 | 0.2827 | 0.5536 | 0.0349 | -0.2786 | 0.3138 |
| **Convex Hull Volume (µm^3^)** | 0.0321 | 0.9132 | 0.4857 | 0.0688 | -0.4071 | 0.1333 |
| **Nº Dendritic Trees** | 0.2668 | 0.3364 | -0.0261 | 0.9264 | -0.0504 | 0.8585 |
| **Maximum Dendritic Order** | -0.0867 | 0.7587 | 0.6676 | 0.0065 | -0.2840 | 0.3049 |
| **Nº of Dendritic Segments** | -0.3038 | 0.2709 | 0.5416 | 0.0371 | -0.3789 | 0.1637 |
|  | **All (n=30)** | | | | | |
|  | **ML** | | **DV** | | **AP** | |
|  | r | p | r | p | r | p |
| **Dendritic Length (µm)** | 0.0394 | 0.8362 | 0.0932 | 0.6229 | -0.0343 | 0.8573 |
| **Convex Hull Volume (µm^3^)** | 0.1439 | 0.4463 | 0.0127 | 0.9476 | -0.0323 | 0.8656 |
| **Nº Dendritic Trees** | -0.0520 | 0.7851 | 0.0823 | 0.6657 | -0.1973 | 0.2959 |
| **Maximum Dendritic Order** | -0.1769 | 0.3497 | 0.3853 | 0.0355 | -0.1261 | 0.5066 |
| **Nº of Dendritic Segments** | -0.3117 | 0.0935 | 0.4166 | 0.0220 | -0.3275 | 0.0773 |
