# Supplementary material for: Dendritic Architecture Predicts in vivo Firing Pattern in Mouse Ventral Tegmental Area and Substantia Nigra Dopaminergic Neurons
Source: Front Neural Circuits. 2021 Nov 19;15:769342. doi: 10.3389/fncir.2021.769342 (PMC8640462; doi:10.3389/fncir.2021.769342)
Supplement: Supplementary file 2 [file Table_2.docx]

Supplementary Material

| **Supplementary Table 2: Absolute and relative dendritic length of SNc and VTA neurons in subdivisions** | | | | | | |  |
| --- | --- | --- | --- | --- | --- | --- | --- |
| **SNc (n=15)** | | | | | | | |
| **Subdivision** | **Nº neurons with dendrites in subdivision** | **Average (µm)*** | **S.e.m** | **Range** | **Average (%)*** | **S.e.m** | **Range** |
| **SNc** | 15 | 1,655 | 140.7 | 866.7-2,701 | 39 | 3.00 | 22-60 |
| **SNr** | 13 | 1,606 | 291.1 | 0-3,406 | 33 | 5.00 | 0-63 |
| **PBP** | 8 | 467.7 | 159.8 | 0-1,912 | 11 | 3.00 | 0-39 |
| **Others** | 13 | 740.4 | 220.6 | 0-3,092 | 17 | 5.00 | 0-68 |
| **VTA (n=15)** | | | | | | | |
| **Subdivision** | **Nº neurons with dendrites in subdivision** | **Average (µm)*** | **S.e.m** | **Range** | **Average (%)*** | **S.e.m** | **Range** |
| **SN** | 10 | 372.8 | 126.8 | 0-1,411 | 7 | 3.00 | 0-32 |
| **PBP** | 15 | 3,859 | 427.2 | 1,478-6,389 | 73 | 4.00 | 37-100 |
| **PIF/PN** | 6 | 367.8 | 176.5 | 0-2,511 | 9 | 4.00 | 0-63 |
| **IF/RLI/CLi** | 5 | 63.53 | 30.48 | 0-351.9 | 2 | 1.00 | 0-9 |
| **Others** | 10 | 411.2 | 110.6 | 0-1,190 | 8 | 2.00 | 0-27 |
|  |  | ***Average values consider both neurons with and without dendrites in the subdivision** | | | | | |
