# Supplementary material for: Dendritic Architecture Predicts in vivo Firing Pattern in Mouse Ventral Tegmental Area and Substantia Nigra Dopaminergic Neurons
Source: Front Neural Circuits. 2021 Nov 19;15:769342. doi: 10.3389/fncir.2021.769342 (PMC8640462; doi:10.3389/fncir.2021.769342)
Supplement: Supplementary file 3 [file Table_3.docx]

Supplementary Material

| **Supplementary Table 3: Spearman correlation values between electrophysiological and cell body position parameters of DA mesencephalic neurons in baseline conditions** | | | | | | |
| --- | --- | --- | --- | --- | --- | --- |
|  | **SNc (n=15)** | | | | | |
|  | **ML** | | **DV** | | **AP** | |
|  | r | p | r | p | r | p |
| **Firing Rate (Hz)** | -0.0140 | 0.9737 | 0.4545 | 0.1404 | -0.6993 | 0.0145 |
| **CV** | 0.2597 | 0.4151 | -0.0982 | 0.7613 | 0.0386 | 0.9052 |
| **CV2** | 0.2448 | 0.4435 | 0.0629 | 0.8517 | 0.1538 | 0.6351 |
| **% Spikes in Burst** | 0.0112 | 0.9724 | 0.1792 | 0.5774 | -0.4777 | 0.1162 |
|  | **VTA (n=15)** | | | | | |
|  | **ML** | | **DV** | | **AP** | |
|  | r | p | r | p | r | p |
| **Firing Rate (Hz)** | -0.0220 | 0.9494 | 0.3187 | 0.2886 | 0.2308 | 0.4478 |
| **CV** | 0.1044 | 0.7370 | -0.6593 | 0.0171 | 0.2033 | 0.5053 |
| **CV2** | 0.0165 | 0.9639 | -0.7143 | 0.0081 | 0.3736 | 0.2094 |
| **% Spikes in Burst** | -0.1713 | 0.5758 | -0.4365 | 0.1359 | 0.6630 | 0.0135 |
|  | **All (n=30)** | | | | | |
|  | **ML** | | **DV** | | **AP** | |
|  | r | p | r | p | r | p |
| **Firing Rate (Hz)** | -0.1938 | 0.3515 | 0.4223 | 0.0365 | -0.3266 | 0.1111 |
| **CV** | 0.4491 | 0.0243 | -0.5457 | 0.0048 | 0.3855 | 0.0570 |
| **CV2** | 0.4047 | 0.0448 | -0.4916 | 0.0126 | 0.4359 | 0.0294 |
| **% Spikes in Burst** | 0.2064 | 0.3223 | -0.1961 | 0.3474 | 0.2489 | 0.2302 |
