# Supplementary material for: Dendritic Architecture Predicts in vivo Firing Pattern in Mouse Ventral Tegmental Area and Substantia Nigra Dopaminergic Neurons
Source: Front Neural Circuits. 2021 Nov 19;15:769342. doi: 10.3389/fncir.2021.769342 (PMC8640462; doi:10.3389/fncir.2021.769342)
Supplement: Supplementary file 4 [file Table_4.docx]

Supplementary Material

| **Supplementary Table 4: Spearman correlation values between morphology and electrophysiological parameters of DA mesencephalic neurons in baseline conditions** | | | | | | | | |
| --- | --- | --- | --- | --- | --- | --- | --- | --- |
|  | **SNc (n=12)** | | | | | | | |
|  | **Firing Rate (Hz)** | | **CV** | | **CV2** | | **% Spikes in Burst** | |
|  | r | p | r | p | r | p | r | p |
| **Dendritic Length (µm)** | -0.0420 | 0.9037 | -0.3719 | 0.2338 | -0.3636 | 0.2463 | -0.4031 | 0.1939 |
| **Convex Hull Volume (mm^3^)** | 0.1329 | 0.6834 | -0.5930 | 0.0421 | -0.4895 | 0.1096 | -0.4703 | 0.1229 |
| **Nº Dendritic Trees** | 0.4957 | 0.1012 | -0.2379 | 0.4565 | -0.1868 | 0.5611 | -0.0288 | 0.9293 |
| **Maximum Dendritic Order** | -0.1096 | 0.7346 | -0.4179 | 0.1764 | -0.4968 | 0.1003 | -0.1248 | 0.6992 |
| **Nº of Dendritic Segments** | 0.2281 | 0.4759 | -0.4349 | 0.1577 | -0.5018 | 0.0965 | -0.0581 | 0.8578 |
|  | **VTA (n=13)** | | | | | | | |
|  | **Firing Rate (Hz)** | | **CV** | | **CV2** | | **% Spikes in Burst** | |
|  | r | p | r | p | r | p | r | p |
| **Dendritic Length (µm)** | 0.1319 | 0.6693 | -0.3846 | 0.1955 | -0.3846 | 0.1955 | -0.1492 | 0.6267 |
| **Convex Hull Volume (mm^3^)** | -0.0495 | 0.8775 | -0.2253 | 0.4590 | -0.2747 | 0.3633 | -0.1215 | 0.6924 |
| **Nº Dendritic Trees** | 0.1379 | 0.6532 | 0.2172 | 0.4760 | 0.2759 | 0.3616 | 0.3925 | 0.1847 |
| **Maximum Dendritic Order** | 0.1834 | 0.5486 | -0.5333 | 0.0605 | -0.5333 | 0.0605 | -0.3689 | 0.2149 |
| **Nº of Dendritic Segments** | 0.2228 | 0.4643 | -0.5750 | 0.0398 | -0.5420 | 0.0557 | -0.4564 | 0.1169 |
|  | **All (n=25)** | | | | | | | |
|  | **Firing Rate (Hz)** | | **CV** | | **CV2** | | **% Spikes in Burst** | |
|  | r | p | r | p | r | p | r | p |
| **Dendritic Length (µm)** | 0.0254 | 0.9048 | -0.3090 | 0.1328 | -0.3131 | 0.1275 | -0.2095 | 0.3148 |
| **Convex Hull Volume (mm^3^)** | -0.0054 | 0.9809 | -0.2755 | 0.1825 | -0.2697 | 0.1924 | -0.2008 | 0.3357 |
| **Nº Dendritic Trees** | 0.2669 | 0.1971 | -0.0234 | 0.9115 | 0.0307 | 0.8840 | 0.1027 | 0.6250 |
| **Maximum Dendritic Order** | 0.0818 | 0.6973 | -0.4057 | 0.0442 | -0.4240 | 0.0347 | -0.2137 | 0.3050 |
| **Nº of Dendritic Segments** | 0.2730 | 0.1868 | -0.5227 | 0.0073 | -0.5379 | 0.0055 | -0.2826 | 0.1710 |
