# Supplementary material for: Dendritic Architecture Predicts in vivo Firing Pattern in Mouse Ventral Tegmental Area and Substantia Nigra Dopaminergic Neurons
Source: Front Neural Circuits. 2021 Nov 19;15:769342. doi: 10.3389/fncir.2021.769342 (PMC8640462; doi:10.3389/fncir.2021.769342)
Supplement: Supplementary file 5 [file Table_5.docx]

Supplementary Material

| **Supplemental Table 5: Spearman correlation values between absolute (µm) or relative (%) dendritic length in different nuclei and electrophysiological parameters of DA mesencephalic neurons in baseline conditions.** | | | | | | | | |  |
| --- | --- | --- | --- | --- | --- | --- | --- | --- | --- |
|  |  |  |  |  |  |  |  |  |  |
| **SNc (n = 12)** | | | | | | | | |  |
| **Subdivision** | **Firing Rate (Hz)** | | **CV** | | **CV2** | | **% Spikes in Burst** | |  |
|  | r | p | r | p | r | p | r | p |  |
| **SNc (µm)** | 0.1538 | 0.6351 | -0.4246 | 0.1689 | -0.3427 | 0.2762 | -0.3247 | 0.3031 |  |
| **%** | 0.0909 | 0.7832 | -0.0140 | 0.9655 | 0.1189 | 0.7162 | -0.0821 | 0.7997 |  |
| **SNr (µm)** | -0.0210 | 0.9483 | -0.6819 | 0.0146 | -0.7180 | 0.0085 | -0.3028 | 0.3386 |  |
| **%** | 0.0701 | 0.8287 | -0.6784 | 0.0153 | -0.7321 | 0.0068 | -0.1907 | 0.5528 |  |
| **PBP (µm)** | -0.3620 | 0.2475 | 0.1948 | 0.5441 | 0.2687 | 0.3984 | -0.2052 | 0.5224 |  |
| **%** | -0.4367 | 0.1558 | 0.3521 | 0.2617 | 0.3583 | 0.2528 | -0.1454 | 0.6520 |  |
| **Others (µm)** | 0.1506 | 0.6403 | 0.3814 | 0.2213 | 0.3678 | 0.2395 | 0.2094 | 0.5170 |  |
| **%** | 0.1296 | 0.6881 | 0.5606 | 0.0579 | 0.5289 | 0.0770 | 0.3963 | 0.2022 |  |
| **VTA (n = 13)** | | | | | | | | |  |
| **Subdivision** | **Firing Rate (Hz)** | | **CV** | | **CV2** | | **% Spikes in Burst** | |  |
|  | r | p | r | p | r | p | r | p |  |
| **SN (µm)** | 0.0000 | 1.0000 | -0.1526 | 0.6187 | -0.1074 | 0.7269 | 0.0682 | 0.8248 |  |
| **%** | -0.0226 | 0.9416 | -0.1300 | 0.6721 | -0.0904 | 0.7689 | 0.0796 | 0.7961 |  |
| **PBP (µm)** | 0.2418 | 0.4258 | -0.5604 | 0.0499 | -0.5275 | 0.0673 | -0.3039 | 0.3128 |  |
| **%** | 0.2766 | 0.3602 | -0.7386 | 0.0039 | -0.7165 | 0.0059 | -0.4645 | 0.1098 |  |
| **PIF / PN (µm)** | -0.4599 | 0.1138 | 0.7287 | 0.0047 | 0.6989 | 0.0079 | 0.3784 | 0.2023 |  |
| **%** | -0.4838 | 0.0939 | 0.7048 | 0.0071 | 0.6630 | 0.0135 | 0.2583 | 0.3942 |  |
| **IF / RLi / CLi (µm)** | -0.2193 | 0.4717 | 0.5482 | 0.0524 | 0.4761 | 0.1000 | 0.2772 | 0.3592 |  |
| **%** | -0.1141 | 0.7106 | 0.3825 | 0.1971 | 0.3288 | 0.2726 | 0.0742 | 0.8096 |  |
| **Others (µm)** | 0.2600 | 0.3910 | 0.0339 | 0.9124 | 0.1696 | 0.5797 | 0.3183 | 0.2893 |  |
| **%** | 0.4186 | 0.1543 | 0.0198 | 0.9488 | 0.1783 | 0.5600 | 0.3643 | 0.2211 |  |
